# Observer-Agent Kinematic Similarity Facilitates Action Intention Decoding

**DOI:** 10.1038/s41598-020-59176-z

**Published:** 2020-02-13

**Authors:** Doriana De Marco, Emilia Scalona, Maria Chiara Bazzini, Pietro Avanzini, Maddalena Fabbri-Destro

**Affiliations:** Consiglio Nazionale delle Ricerche (CNR), Istituto di Neuroscienze, sede di Parma, Italy

**Keywords:** Cognitive neuroscience, Motor control, Sensorimotor processing, Social behaviour

## Abstract

It is well known that the kinematics of an action is modulated by the underlying motor intention. In turn, kinematics serves as a cue also during action observation, providing hints about the intention of the observed action. However, an open question is whether decoding others’ intentions on the basis of their kinematics depends solely on how much the kinematics varies across different actions, or rather it is also influenced by its similarity with the observer motor repertoire. The execution of reach-to-grasp and place actions, differing for target size and context, was recorded in terms of upper-limb kinematics in 21 volunteers and in an actor. Volunteers had later to observe the sole reach-to-grasp phase of the actor’s actions, and predict the underlying intention. The potential benefit of the kinematic actor-participant similarity for recognition accuracy was evaluated. In execution, both target size and context modulated specific kinematic parameters. More importantly, although participants performed above chance in intention recognition, the similarity of motor patterns positively correlated with recognition accuracy. Overall, these data indicate that kinematic similarity exerts a facilitative role in intention recognition, providing further support to the view of action intention recognition as a visuo-motor process grounded in motor resonance.

## Introduction

When we observe another individual performing an action, several aspects concur to inform us about not only what action is taking place, but also which motor intention is driving its performance. A major role in these cognitive processes is played by the motor system^1^. Imagine observing someone grasping and lifting a glass: the visual information flows from visual to temporal areas devoted to biological motion perception, and from there, it reaches and activates the same motor fronto-parietal networks recruited during the execution of the same action^2^. Named mirror mechanism, this mechanism allows us to have an automatic and internal representation of the observed actions which matches our motor repertoire^3–5^. At the same time, most of the skilled actions we execute in our everyday life are composed of several motor acts (in the example above one has to reach the glass, grasp, and finally lift it), and the way these are chained together would contribute information about the motor intention underlying that specific action^6^. The picture described above provided the physiological counterpart of the view that the process of motor intention recognition is mediated by the same mechanisms that govern the motor control of intentional actions^7^.

At the same time, parallel aspects contribute information about the intention underlying an observed action. On one side, contextual elements are fundamental cues for decoding motor intentions. Grasping and lifting a glass in the middle of a party would likely signal the intention to cheer with friends, while the same action performed at the table of a restaurant with an empty glass could signal the request to have another glass. On the other side, a great interest emerged around how the kinematics of the observed action could be by itself a cue of the motor intention. Indeed, while several studies proved how the kinematics of reach-to-grasp movements is affected by specific variations of factors like object position, type, size or familiarity^8–13^, kinematics modulations of reaching and grasping were found even when the same object was grasped with different intents, as for example to place or to throw it in a container^14,15^. Moreover, further modulations emerged associated to social intentions, as in the case of a movement addressed toward another individual in opposition to ourselves^16–20^, or when it has cooperative or competitive goals^21–23^. In turn, the kinematics of the observed action can modulate the reactivity of the observer’s motor system^24–29^.

Despite the large amount of studies, it is still a matter of debate which kinematic parameters of reach-to-grasp movements are more strongly modulated by underlying motor intention. A recent meta-analysis conducted by Egmose & Køppe^30^ showed that changes in both target properties (intrinsic/extrinsic features) and prior intentions affect both the reach and the grasp components (see also^31^). While the maximal finger aperture during reaching is the parameter most sensitive to the variation of intrinsic object properties, and especially its size, a variation of reach duration and deceleration timing emerged in response to tasks with different accuracy requirements (i.e., grasping an object to place it carefully as opposed to throwing it). This was in line with the “precision hypothesis” formulated by Marteniuk and colleagues^15^ proposing that a higher level of precision and accuracy of the action would be associated to a longer deceleration phase of the movement. Similarly, when the action develops in a social context, the interaction with another individual results in longer reaching duration with respect to the individual (i.e. non-social) execution^16–20,32^.

The knowledge that different motor intentions modulate the kinematics of human behavior opens to the possibility that purely the observed kinematics may serve as a cue to predict others’ intentions. Manera and colleagues^33^ demonstrated that it is possible to predict the agent’s intention to cooperate or compete with a partner simply by observing the initial reach and grasp phase of the entire action, in the absence of any contextual information. Similar results were found by Sartori *et al*.^22^, while Naish *et al*.^34^ failed to report a capacity to discriminate grasp-to-place vs. grasp-to-eat intentions even if the actions significantly differed in terms of kinematics features.

A recent study by Cavallo *et al*.^35^ evidenced a significant relation between the accuracy in an intention prediction task and how much a specific subset of kinematics features was recognizable as varying across different intentions. In this study, the authors recorded video clips and kinematics of a set of grasping movements performed by seventeen different agents with two different intentions (i.e., grasp a bottle to drink or to pour). A subset of kinematics features that significantly varied across the different intentions were then identified by mean of a linear discriminant analysis (LDA): the results were used as criteria to select the video clips which expressed significant variations of the discriminative kinematics variables. The selection of video stimuli was further manipulated in order to maximize or minimize the visibility of the kinematics variations, causing a significant increase/decrease in intention prediction performance. In other words, since different intentions are represented by different kinematic patterns, if the expressed movement variation is higher, the resulting intention-discrimination task becomes easier.

Given these premises, an open question is whether the capability to decode others’ intentions on the basis of their kinematics depends solely on how much this kinematics varies across different actions, or rather it is also influenced by the similarity with the observer motor repertoire. Indeed, the “matching” between the other/own motor repertoires in terms of low-level kinematic features might be a crucial factor underpinning our capability of intention understanding.

Previous studies have already demonstrated that observing motorically “familiar” actions relative to the own motor expertise determined a greater mirror motor activation (i.e., stronger motor resonance) and finer action prediction capability with respect to what happens while observing “unfamiliar” actions^36,37^. These findings suggest that motor resonance is a mechanism favoring action prediction, in particular in case of familiar action well represented in the observer’s motor repertoire. However, little is known about how differences and similarities in terms of kinematic features of the movement can influence action intention recognition.

In the present study, we investigated the role of kinematics similarity between the agent and the observer in decoding the motor intention of an action. To this aim, we performed a kinematic study including two tasks on the same group of subjects. In the first, participants were asked to perform a reach-to-grasp and place action, which could be embedded in social or non-social contexts. In the non-social context, participants had to put the object in a big or small box, which required different precision (see^15,38^); in the social context they had to give the object to another individual with hands mimicking the position and size of the boxes.

The same group of participants underwent a second experiment in which they were asked to observe and recognize the motor intention of an actor executing one of the actions previously performed. During the observation, the placing phase was visually-occluded requiring the participant to decode the intention from the observation of the sole reach-to-grasp phase.

The similarity between the kinematics profiles of each participant and the actor was assessed, and its possible effects on the recognition accuracy were evaluated. Our hypothesis was that the kinematic similarity could positively affect the accuracy in intention recognition.

## Methods and Results

### Participants

21 naïve volunteers (6 males and 15 females, mean age 24.8 ± 0.6) took part in experiment 1 (execution task). The second part of the study, namely the experiment 2 (observation task), was administered to the same group of subjects immediately after the conclusion of experiment 1. The minimum sample size was defined on the basis of previous kinematic studies which investigated the modulation of reach-to-grasp kinematics in response to different motor intentions^22,33,35,39^.

All participants were right-handed (according to Edinburgh Handedness Inventory^40^). They had normal or corrected-to-normal vision and no history of neurological or psychiatric disorder.

The study was approved by the local ethical committee (Comitato Etico Unico per la Provincia di Parma) and was conducted according to the principles expressed in the Declaration of Helsinki. The participants provided written informed consent.

## Experiment 1 (Execution Task)

### Apparatus, stimuli, and procedure

**Figure 1:**
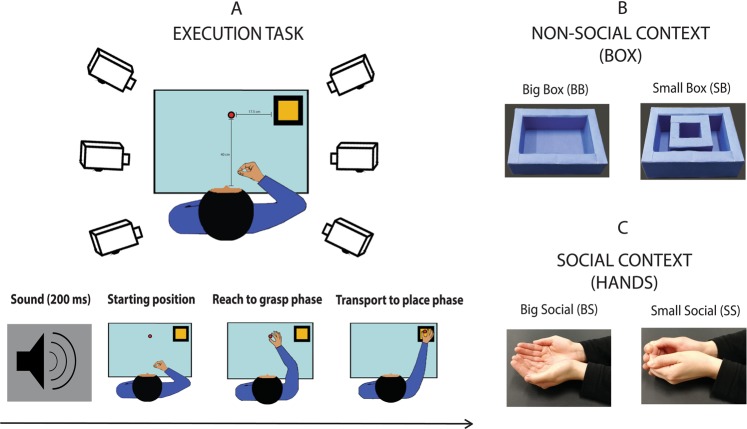
The experimental setting of execution task is presented in panel A – upper part. In the lower part, the timeline of an experimental trial is presented, depicting in sequence: go-signal, initial starting Position (SP), the end of the reach to grasp movement toward the object and the final placing phase of the action. Panel B and C depict the different targets presented in each experimental condition.

Participants (Fig. 1A) sat comfortably in front of a table, keeping their right hand with the thumb and index finger set in a pinching position (starting position, SP). The SP was located 15 cm to the right of the participant’s mid-sagittal plane and 20 cm from his chest. A small cylindrical object (3 cm diameter) was positioned on the table at a distance of 40 cm from the chest. The monitor of a PC (19-inch LCD) was placed on the table plane 90 cm from participants’ forehead.

Participants were required to reach and grasp the object (reach-to-grasp phase), and then to transport and place it into a specific target container (place phase). The final target was manipulated by considering four experimental conditions presented in two different blocks. In the first (Non-social context, Fig. 1B), the subject placed the cylinder in a box that could be big or small (16 × 11.2 × 4 cm; 7.3 × 6.5 cm × 4 cm, respectively). In the second (Social context, Fig. 1C), participants were requested to put the object into the hands of a human agent seated at the right side of the table. The agent’s hands were resting on the table, kept opened and supine so to match the big and small boxes in terms of position and size.

Each target (box or hands) was presented separately and their location was identical in all the conditions (17.5 cm at the right side of the object). In both blocks, participants were asked to return in the SP after the action was accomplished.

At the beginning of each run (i.e., a subgroup of trials), a written instruction indicating the final target of the action was presented. An acoustic tone (200 ms duration) signaled to start the movement. Participants performed 10 trials for each target [Big Box (BB), Small Box (SB), Big Social (BS), Small Social (SS), Fig. 1B,C]. As only one target had to be present on the table during the execution, an experimenter was in charge of switching the target object. Trials were further grouped in 8 runs (each with 5 trials with the same target), whose order was randomized and counterbalanced across participants.

### Movement recording and data analysis

The movements of the participants’ right arm were recorded using the 3D-optoelectronic SMART system (BTS Bioengineering, Milano, Italy). This system consists of six video cameras detecting the position of 4 infrared reflecting markers (6 mm or 10 mm diameter spheres) at a sampling rate of 120 Hz. The spatial resolution was 0.3 mm.

The 4 markers were positioned on subject’s right arm as follows:

- 1 marker on the elbow in correspondence of the lateral epicondyle of the humerus.
- 1 marker in correspondence to the radius-styloid process of the wrist.
- 1 marker on the thumb nail.
- 1 marker on the index finger nail.

The reaching kinematics was monitored by recording time-wise the trajectory of the wrist marker. Moreover, the vertical displacement between the wrist and the elbow markers was calculated.

The grasping kinematics was monitored by recording time-wise the distance between the thumb and index markers. The prototypical grasping movement is supposed to contain an initial phase of finger opening, ending when this aperture peaks (maximal finger aperture), followed by a phase of finger closing onto the object^41^.

Data were analyzed using homemade code developed in MATLAB (R2016b). The analysis focused on the reach and grasp phase, excluding the placing one, in order to measure any difference in that part of the action that was common to all the conditions unless for the different “intentional” component. A Gaussian low pass smoothing filter (sigma value: 0.93) was applied to the recorded data.

The beginning of the reaching was chosen as the first of at least three consecutive frames during which the displacement of the wrist marker along any Cartesian body axis increased more than 0.3 mm with respect to the previous frame. To determine the end of the reaching, we calculated the endpoint separately along the X, Y, and Z directions. For each axis, we requested that 3 consecutive time frames showed a displacement lower than 0.3 mm with respect to the previous frame. As slight differences were found among results for X, Y and Z directions, the global endpoint was selected as the one closer to the grasping end^32^.

The beginning of the grasping was considered as the first of three consecutive frames in which the distance between the two markers placed on the right fingers increased more than 0.3 mm with respect to the previous frame. The end of the grasping was considered as the first of at least three consecutive frames after the beginning of finger closing in which the distance between the two fingers decreased less than 0.3 mm with respect to the previous frame.

In order to investigate how different motor intentions affected the reaching and grasping movements, a series of kinematic parameters was considered, listed in Table 1. We computed the following variables:

- Reach Amplitude (RA), defined as the length of the trajectory of the wrist marker (mm).
- Reach Elevation (R_y_), defined as the vertical (Y) projection of the wrist marker trajectory (mm).
- Reach Curvature (R_z_), defined as the left-right (Z) projection of the wrist marker trajectory (mm).
- Reach Velocity (RVel), defined as the magnitude of the velocity vector of the wrist marker (mm/s).
- Reach Acceleration (RAcc), defined as the derivative of the wrist velocity calculated during the acceleration phase, i.e., between the reaching start and the peak of velocity of the wrist (mm/s^2^).
- Reach Deceleration (RDec), defined as the derivative of the wrist velocity calculated during the deceleration phase, i.e., between the peak of velocity of the wrist and the end of the reaching (mm/s^2^).
- Vertical Wrist-Elbow Distance (WE_Y_), calculated as the vertical (Y) projection of the distance between the wrist and the elbow markers (mm). The increase or decrease of the WE_Y_ during reaching indicated a forearm rotation toward the left of the right side.
- Grasp Aperture (GA), defined as the distance between the fingers markers (mm).
- Grasp Velocity (GVel), defined as the velocity of fingers aperture and closure (mm/s).

**Table 1:** List of the reaching and grasping kinematic parameters assessed in experiment 1. Crosses indicate values calculated for each parameter. Maximum (peaks) and ROM values were calculated both for trajectory and derivative measures (i.e., velocity and acceleration). For the latter, mean values were also considered.

Reaching kinematic parameters								Grasping kinematic parameters	
	Trajectory Amplitude(mm)	Elevation (mm)	Curvature (mm)	Wrist-Elbow Distance (mm)	Velocity (mm/s)	Acceleration (mm/s^2^)	Deceleration (mm/s^2^)	Aperture (mm)	Velocity (mm/s)
Mean					X	X	X		X
Maximum		X	X	X	X	X	X	X	X
ROM		X	X	X	X	X	X	X	
Length	X								

For each parameter, we calculated the peak value and the Range of Motion (ROM, calculated as the difference between the maximum and the minimum value). For the velocity parameters we also calculated the average values.

### Statistical analysis

A 2 × 2 repeated-measures ANOVA was carried out on each of the aforementioned parameters with CONTEXT (No-Social/Social) and SIZE (Big/Small) as within-subject factors. All variables were normally distributed as verified by Kolmogorov-Smirnov Test (p > 0.05). Partial η^2^ was calculated as a measure of effect size.

## Experiment 2 (Observation Task)

### Apparatus, stimuli and procedure

Participants sat comfortably in front of a table, with their right hand positioned on a keyboard. The monitor of a PC (19-inch LCD) was placed on the table plane, 60 cm distant from the participant’s forehead.

**Figure 2:**
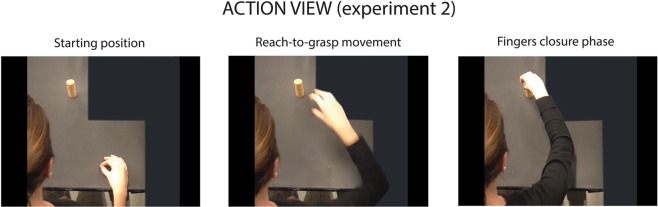
Frames (initial, middle and final) extracted from an exemplar video stimulus presented during the observation task. The action was shown from a perspective resembling an egocentric point of view. Each video started with the actress still with the hand and fingers in a pinch position and ended at the hand-object contact.

Stimuli consisted of video clips showing an actor executing the same reach, grasp and place actions performed by the participants in the Experiment 1. The actions were recorded using a high definition camera positioned in order to provide a slightly right-sided top view of the actor (Fig. 2). We chose to film and present the stimuli from an egocentric perspective in order to maximize a potential motor resonance effect (see^42^).

The actor was required to perform 10 repetitions of the actions used in the experiment 1 [Big Box (BB), Small box (SB), Big Social (BS), Small Social (SS)]. The kinematics of the actor movements was recorded using the same setup as in experiment 1.

Video clips were edited masking the target of the action, in order to exclude any contextual cue that could reveal the actor intention (Fig. 2).

Stimuli presentation and randomization procedures were controlled using Psychtoolbox-3^43–45^. Videos were presented in three different blocks where conditions were coupled two by two:

1. *Big Box vs. Small Box* (BB-SB). In this block, participants were asked to classify whether the observed reach and-grasp actions had been performed before placing the object in the big or in the small box. We tested the predictability of the factor SIZE on the basis of the observed kinematics regardless of the factor CONTEXT.
2. *Big Box vs. Big Social* (BB-BS). In this block, participants were asked to classify whether the observed reach and-grasp actions had been performed before placing the object in the big box or into the hands of the individual. We tested the predictability of the factor CONTEXT while maintaining the dimension of the targets (Big) fixed.
3. *Small Box vs. Small Social* (SB-SS). In this block, participants were asked to classify whether the observed reach and-grasp actions had been performed before placing the object in the small box or into the hands of the individual. We tested the predictability of the factor CONTEXT while maintaining the dimension of the targets (Small) fixed.

We decided to test twice the factor CONTEXT (BB-BS and SB-SS) since we had no *a-priori* hypothesis favoring the test for small or big conditions. Conversely, considering the previous literature about the kinematic modulation of factor SIZE^9,12,13,15,30^ we judged it redundant to re-test it in the social context.

Each trial started with the presentation of a fixation cross (500 ms duration) followed by the video clip showing the action performed by the actor. Regardless of the final goal, only the first part of the movement (reach and grasp phase) was shown. The video clip was then freezed at the first frame depicting finger object contact (Fig. 2).

Participants were requested to carefully observe the movement and predict the final target of the action by pressing the left or right arrow keys with the right index or middle finger; key assignment was counterbalanced between subjects. The video remained frozen until the participant responded. Participants were instructed to respond as accurately and as quickly as possible.

Each block consisted of 40 trials (20 for each condition) presented in a pseudorandom order, for a total of 120 trials. Blocks presentation order was randomized and counterbalanced between participants. No feedback was given to the participants, except in 10% of total trials (pseudo-randomized and balanced across conditions) where the final part of the action was shown without masking of the target. A brief series of 8 practice trials was administered before the session.

### Behavioral data analysis

Participants’ prediction accuracy was assessed. In addition, the timing of the response (RTs) was collected and compared with the video clip duration in order to consider only responses given after the observation of the whole reach-to-grasp action. Moreover, only the trials with RTs values that lied within ±2 SD from the subject mean were included in the statistical analysis. Only one subject was excluded from data analysis because of a too high number of responses given before the end of the actor movement.

Since general accuracy rate in a binary task can represent a biased measure of accuracy (Signal Detection Theory, SDT^46,47^; see also^22^), we also calculated the proportion of correct hits (i.e., when the subject correctly identified the intention) and false alarms (i.e., when the subject erroneously recognized an intention that was not present). The proportion of hits and false alarms was used to calculate SDT parameters as the *c* criterion and *d*′ sensitivity index, adopting the correction in case of extreme false alarms (0% accuracy) or hits (100% accuracy) proportions^48^. The c criterion represents the subject tendency to respond in favor to a condition respect to another and represented a quantitative measure of a biased decisional criterion (a value of 0 represent a neutral bias). The *d*′ is an unbiased quantitative measure of the ability to distinguish the two conditions (higher values represent higher ability of discrimination).

### Kinematic similarity analysis

The same reach and grasp kinematic parameters measured in experiment 1 were calculated from the actor movements and analyzed with the same procedure and statistical methods (see Data Analysis of experiment 1). This was done in order to compare the kinematics of the actor (i.e., the observed kinematics in experiment 2) with the kinematics of each participant (i.e., the performed kinematics in experiment 1). This comparison allowed us to investigate the role of the similarity between the executed/observed kinematics profiles across conditions in action intention recognition. Mean values and Standard errors of the reference subject and participants’ kinematic parameters are reported in Supplementary material (S1-S2).

The Linear Fit Method (LFM^49^), complemented with the Root Mean Square Error (RMSE^50^), were selected to assess the waveform curve similarity^51^. LFM calculates the linear regression between the dataset under investigation, returning information about the scaling factor (a_1_), the weighted average offset (a_0_), and the trueness of the linear relation between them (R^2^). When the curves under analysis are equal, the values of LFM parameters tend to their ideal values, i.e. a_1_ = 1, a_0_ = 0 and R^2^ = 1. In particular, we considered the determination coefficient R^2^ which is a measure of the similarity, especially for the temporal pattern, between the compared curves. When R^2^ > 0.5 the assumption of linearity is considered as valid and the curves could be considered similar. Higher R^2^ values indicate higher similarity. In order to have a measure of data dispersion we selected the RMSE. RMSE represents the root square of the variance, evaluated sample by sample, between the curves under analysis. RMSE values increase when the variability among the curves increases. Supplementary Figure S3 shows three example cases of R^2^ and RMSE values with different combinations of curves under investigation. In particular, in panel a, the curves have a similar temporal pattern that leads to high R^2^ value, and low dispersion with consequently a low RMSE value. Panel b shows curves with a similar temporal pattern (high R^2^) but high dispersion between curves (high RMSE). In Panel c the worst case is presented: the curves have non-similar temporal pattern with a consequent low value of R^2^ and high data dispersion with a consequent high value of RMSE.

The similarity analysis was performed on the kinematic curves relative to the parameters that showed a significant difference in the experiment 1, i.e. RA, RVel, RAcc, RDec, GA, GVel. All curves were normalized within participants in order to be matched in duration. The dataset for each subject was obtained considering all the possible combinations based on the number of the valid trials per condition of the given subject. Conditions were concatenated two by two according to the scheme of experiment 2 (BB-SB, BB-BS, SB-SS). More specifically, if the *i-th* subject has a number of valid trials equal to N for the first condition and M valid trials for the second condition, we recreated a total of N × M concatenated curves. The same procedure was applied on the curves of the reference subject (N′ × M′). Once the two datasets were obtained, similarity indices (R^2^ and RMSE) were computed for all the possible combinations (N × M) × (N′ × M′) of the concatenated curves of the *i-th* subject and of the reference subject.

Finally, the average R^2^ and RMSE were calculated for each subject. The output of this analysis provides a dual information: on one side how much the curves are different across conditions and within subject; on the other side, how much the concatenated curve of each participant is similar to that of the actor.

### Statistical analysis

Average *d*′ scores were submitted to a univariate ANOVA with CONDITION (BB-SB, BB-SB, SB-SS) as within-subject factors. An additional series of one-sample t-tests was performed on c values in order to check for the presence of a decision bias within each condition. Sphericity of the data in the ANOVA was verified before performing the statistical analysis (Mauchly’s test, p > 0.05). All variables were normally distributed as verified by Kolmogorov-Smirnov Test (p > 0.05). Partial η^2^ was calculated as a measure of effect size.

A Pearson’s correlation was calculated between similarity indices (R^2^ and RMSE) and behavioral accuracy (*d*′ scores), in order to measure the relationship between the reach-to-grasp features (kinematics variations) and the performance in intention recognition. The p-values resulting from the correlation analysis were corrected for multiple comparison by using the FDR method^52^. In addition, we computed the Bayes factor for each of the correlations resulting significant at an uncorrected level. The Bayesian statistics were implemented to offer a measure of probability of the real contribution of kinematic similarity on intention recognition accuracy. Indeed, the sole p-value gives us the probability of obtaining the observed data assuming that H0 is true (i.e., that no relation between similarity and intention recognition accuracy exists), but it does not tell us anything about how likely it is that H1 is true.

## Results

### Experiment 1 (execution task)

#### Kinematic results

**Figure 3:**
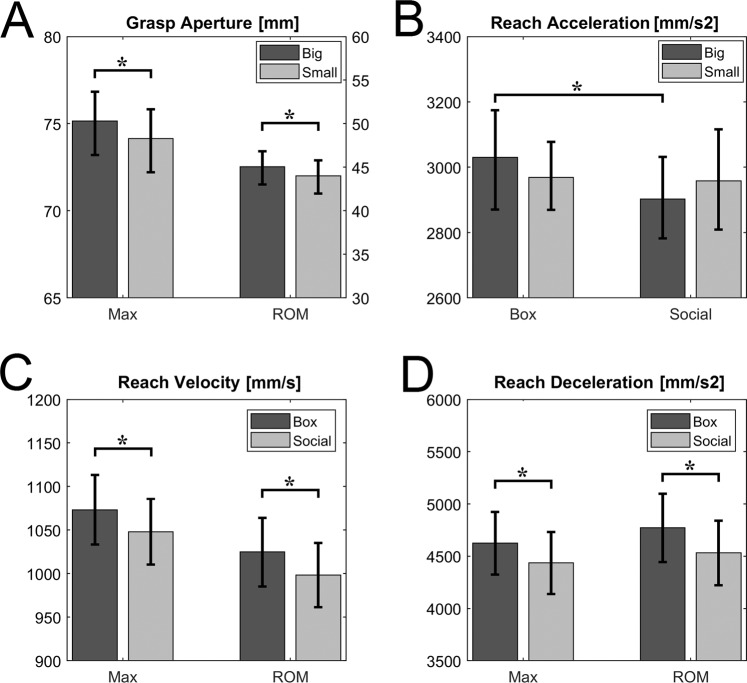
Panel A reports the variables whose main effect SIZE resulted significant for both max values and range of motion. Left Y axis refers to max values, the right one to ROM values. Panels C and D show the variables whose main effect CONTEXT resulted significant for both max values and range of motion. Asterisks indicate a significant p-value at the ANOVA main effect (p < 0.05). Panel B shows the significant SIZE × CONTEXT interaction effect relative to the Reach Acceleration Mean. Asterisk indicates the significant post-hoc comparison between Big Box and Big Social (p < 0.05).

Main significant results concerning Reach and Grasp parameters are reported in Fig. 3. The ANOVAs showed a significant main effect of SIZE on RA (*F(1*,*20)* = 4.87, *p* = 0.04, *η*^2^*partial* = 0.20), GA peak ( *F(1,20) *= 4.14, *p* = 0.05, *η*^2^*partial* = 0.17, Fig. 3A), GA ROM (*F(1*,2*0)* = 4.51, *p* = 0.04, *η*^2^*partial* = 0.18, Fig. 3A) and GVel Mean (*F(1*,2*0)* = 4.70, *p* = 0.04, *η*^2^*partial* = 0.19). In summary, both reaching and grasping movements were affected by target size. The reach trajectory was higher when the participants had to place the object in a big target compared to a small target, independently from the social/non-social context. Similarly, finger aperture resulted wider and faster when participants had to put the object in big targets (box or hands) respect to the smaller ones.

Concerning the CONTEXT factor, we found a significant main effect on RVel Peak (*F(1*,2*0)* = 4.62, *p* = 0.04, *η*^2^*partial* = 0.19, Fig. 3C), RVel ROM (*F(1*,*20)* = 4.96, *p* = 0.04, *η*^*2*^*partial* = 0.20, Fig. 3C), RDec Peak (*F(1,20)* = 4.66, *p* = 0.04, *η*^*2*^*partial* = 0.19, Fig. 3D) and RDec ROM (*F(1,20)* = 4.40, *p* = 0.05, *η*^*2*^*partial* = 0.18, Fig. 3D). These results showed that when the task afforded a social intention (i.e., participants were required to put the object in the hands of a person), the reaching was slower compared to the no-social context, regardless of the target size. This is also in line with the lower deceleration found during the object approaching.

Moreover, we found a significant CONTEXT × SIZE interaction effect on RAcc Mean (*F(1,20)* = 4.53, *p* = 0.05, *η*^*2*^*partial* = 0.18, Fig. 3B). Post-hoc analysis performed with Neuman-Keuls test evidenced a significant contrast between BB and BS conditions (*p* = 0.02), evidencing that the arm acceleration was lower in social vs. non-social contexts, but only limiting the comparison to big sized targets.

### Experiment 2 (observation task)

#### Behavioral results

The ANOVA on *d*′ scores evidenced a significant effect of factor CONDITION (*F(2,18)* = 28.12, *p* < 0.001, *η*^*2*^*partial* = 0.58). Post-hoc analyses showed that all the comparisons were significant [BB-SB (no-social) vs. BB-BS (social, big dimension), *p* = 0.0001; BB-SB (no social) vs. SB-SS (social, small dimension), *p* = 0.002; BB-BS (social, big dimension) vs. SB-SS (social, small dimension), *p* = 0.001]. Participants were significantly more accurate in predicting action intention in the BB-SB condition (correct responses percentage: 85%, *d*′ = 2.40), followed by SB-SS (73%, *d*′ = 1.45) and BB-BS conditions (58%, *d*′ = 0.41). The *d*′ scores were tested against zero (chance level) by means of one sample t-tests. *d*′ were largely significantly different from zero for the first two condition (*p* < 0.0001), while significance was weaker for the last one (*p* = 0.02), in line with the lower and near to the chance level accuracy rate.

One sample t-tests on *c* values resulted not significant for all the conditions (*p* > 0.05). This result evidenced that no significant decision bias were present during the task.

### Relation between kinematics and behavioral performance

**Figure 4:**
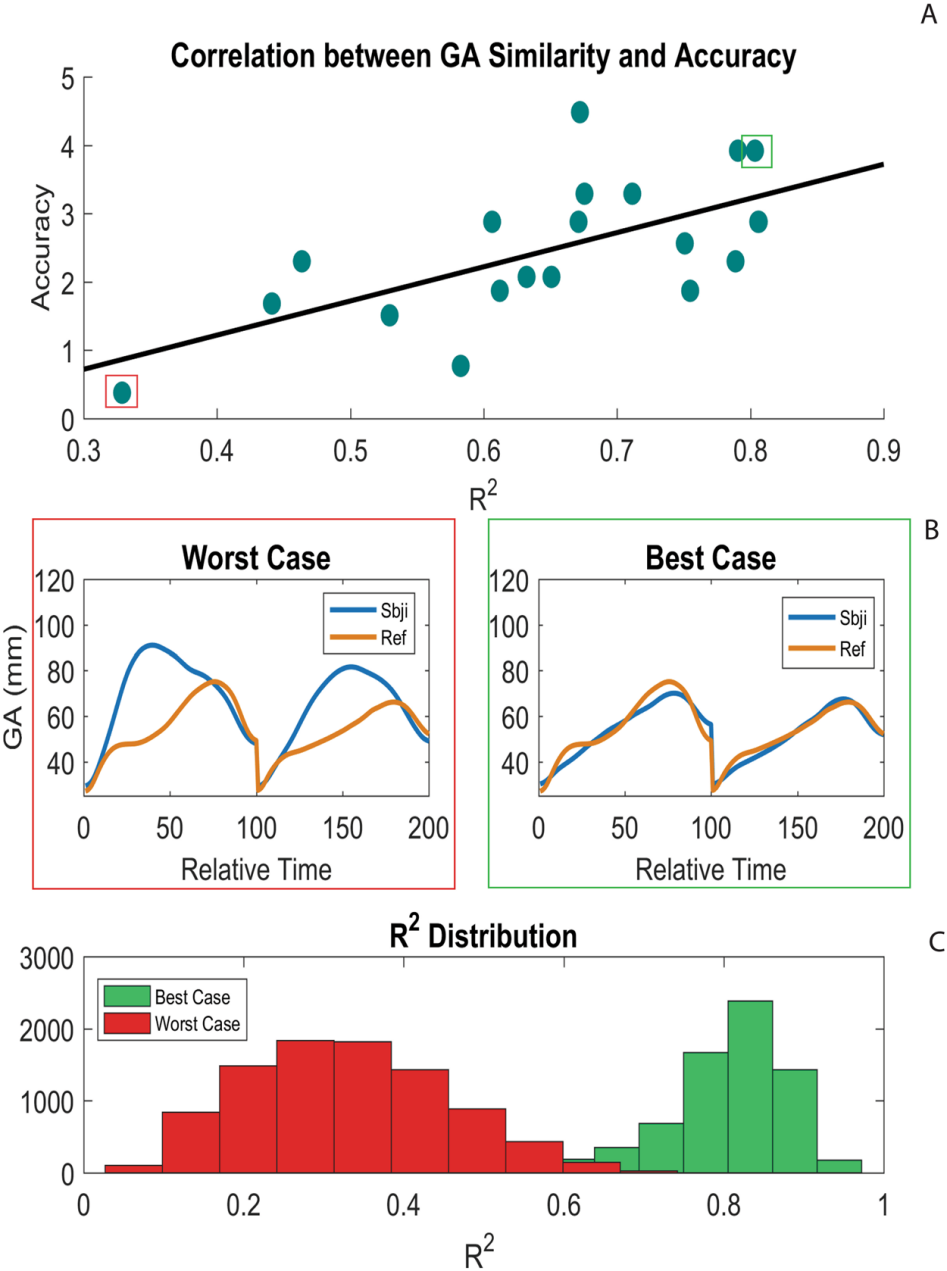
Similarity-Accuracy correlation (r = 0.65) relative to the grasp aperture in the non-social condition (Big Box vs. Small Box). (**A**) Blue dots indicate [R^2^-accuracy] for each subject, while black line indicates the linear trend of this distribution (y_accuracy_ = 5.00(x_SI_) − 0.80). (**B**) For two representative subjects (worst and best cases in terms of accuracy), the average kinematic curves of the participant (blue line) and of the reference subject (orange line) are shown. (**C**) R^2^ distributions among all individual trials are reported for the worst (red bars) and the best (green bars) subjects.

Main significant correlation results are reported in Table 2. Considering the BB-SB contrast, total accuracy (*d*′ scores) showed a significant positive correlation with GA R^2^ (*r* = 0.65, *p* = 0.03, Fig. 4), while negative relationships appeared with GA RMSE (*r* = −0.44, *p* = 0.16) and GVel RMSE (*r* = −0.44, p = 0.16), though only significant at an uncorrected level.

**Table 2:** Summary of the main results of the Bayesian correlation analysis between accuracy and similarity indexes for each kinematic parameter for Non-social (BB-SB) and Social (BB-BS) contrasts.

Correlation Pair	r	corrected p-value	BF_10_	BF Robustness Check
GA_R^2^– BB-SB Accuracy	0.65	0.03	25.03	Strong
GA_RMSE – BB-SB Accuracy	−0.44	0.18	1.67	Anecdotal
GVel_RMSE – BB-SB Accuracy	−0.44	0.18	1.69	Anecdotal
RDec_RMSE – BB-BS Accuracy	−0.47	0.18	2.27	Anecdotal
RAcc_RMSE – BB-BS Accuracy	−0.55	0.08	5.68	Moderate

**Figure 5:**
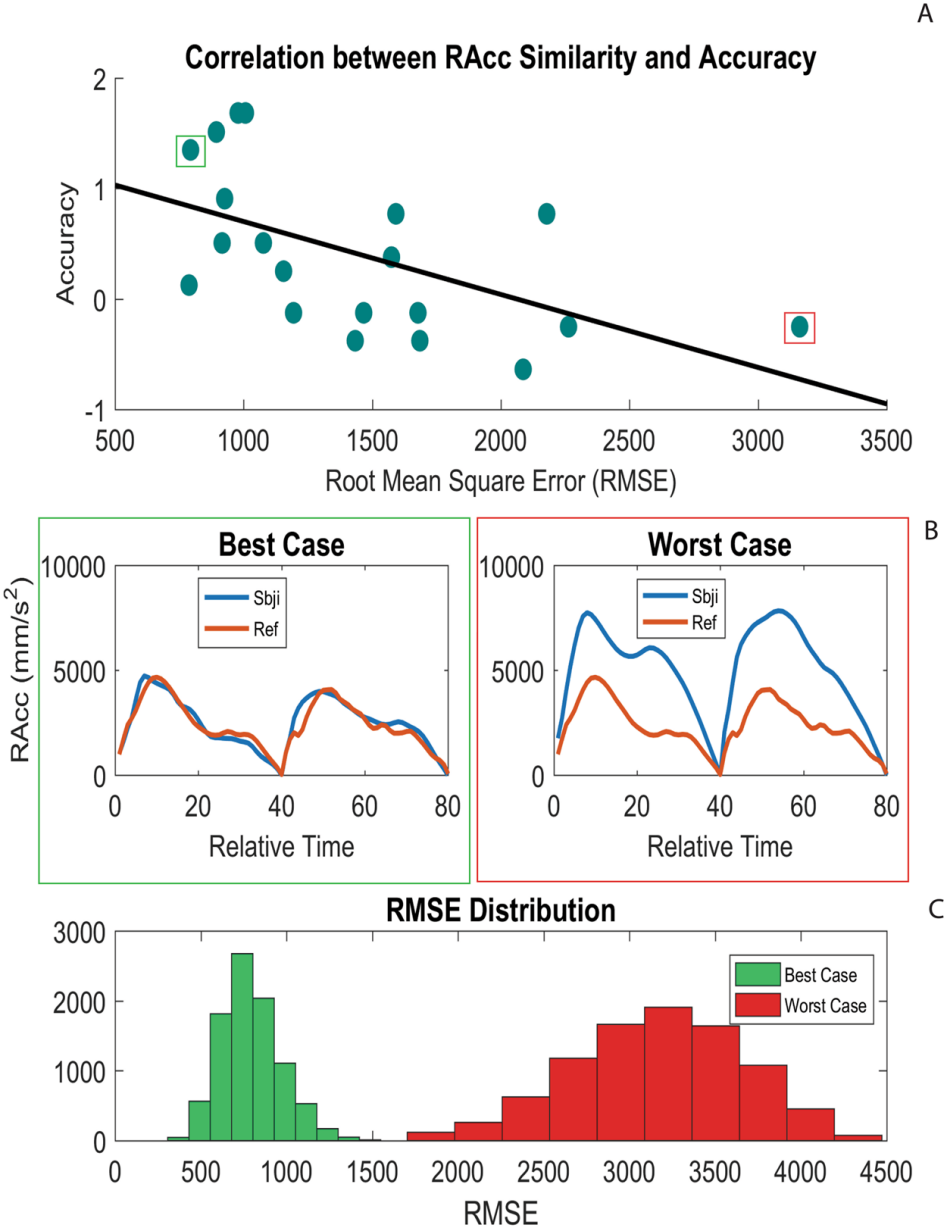
RMSE-Accuracy correlation (r = −0.55) relative to the reach acceleration in the social condition (Big Box vs. Big Social). (**A**) Blue dots indicate [RMSE-accuracy] for each subject, while black line indicates the linear trend of this distribution (y_accuracy_ = −0.0006(x_RMSE_) + 1.36). (**B**) For two representative subjects (worst and best cases in terms of accuracy), the average kinematic curves of the participant (blue line) and of the reference subject (orange line) are shown. (**C**) RMSE distributions among all individual trials are reported for the worst (red bars) and the best (green bars) subjects.

Concerning the BB-BS contrast, no significant correlation emerged between total accuracy and any R^2^ index. However, a negative correlation with a trend to significance was found between total accuracy and RAcc RMSE (*r* = −0.55, *p* = 0.08, Fig. 5). A negative correlation appears also with RDec RMSE (*r* = −0.47, *p* = 0.16), which resulted significant only at an uncorrected level. No significant results emerged concerning the SB-SS contrast.

The Bayes Factors (BF_10_, Table 2) confirmed that the hypothesis of a significant correlation between similarity indexes (R^2^ and RMSE) and intention recognition accuracy is better supported by the data than the alternative (no relation). The BF robustness check (see^53^) indicated strong evidence for GA R^2^ (H1 is likely to occur 25.03 times than H0) and moderate evidence for RAcc RMSE (H1 is likely to occur 5.68 times than H0).

Summarizing the results, the similarity between the executed/observed actions had a meaningful contribution in facilitating motor intention prediction when participants had to discriminate between two goals based on size variation (non-social condition). Specifically, the more similar their own and the observed hand shaping, the higher was the prediction accuracy in the observation task (see Fig. 4).

Similar but weaker results were found when participants had to discriminate between social and non-social intentions. The arm acceleration appeared to play a key role in this contrast, being modulated in social vs. non-social actions, and its similarity between the actor and the observer was linked to the accuracy in intention prediction. Here it has to be noted that the reported features have not to be intended as universal (e.g. lower arm acceleration does not necessarily characterize all social actions), but their value -restricted to the contrasts under examination – lies in the reliable parallelism between execution and performance in intention discrimination.

The result on arm acceleration was found only for one of the two tasks requiring a recognition of a social intention, i.e., the BB-BS condition. Considering that accuracy in the SB-SS contrast was significantly above chance (73%), we thus wondered whether participants could have used cues other than the reach-to-grasp kinematics to accomplish correctly the task. Having already analyzed all the kinematic variables proper of the reach-to-grasp movement, and without any contextual element that could inform about the to-be-discriminated conditions, we thus hypothesized the presence of a spatial cue, i.e. the orientation of the hand while grasping the object. In other words, a peculiar hand configuration at the end of the grasping phase might have worked out as an additional spatial cue to discriminate the final intention of the action.

### Relation between hand orientation and behavioral performance

To quantify this aspect, we considered the triangle formed by the wrist, thumb and index markers, and projected it onto the three anatomical planes separately (sagittal, frontal, transversal). For each subject and condition, we computed the area of the three projections at the exact time frame of hand-object contact (i.e., grasping end). Supposing that the object could have been grasped with different orientations, this should reflect in a modulation, between conditions, of at least one of these components (see Supplementary Fig. 4A).

Since the object prehension mostly occurred in the sagittal and transversal planes, only the projections of the areas calculated for these two planes were considered. Then, the variations expressed in percentages (Δ_S_ and Δ_T_) were calculated comparing the contrasts presented in experiment 2 (BB-SB, BB-BS, SB-SS). One sample t-tests evidenced no significant differences for all planes, except for the transversal one (*t(20)* = 2.03; *p* = 0.05) in SB-SS contrast.

The area of the projection on the transversal plane significantly increased when the action required to put the object in the small box compared to the hands, suggesting that participants used a more lateral prehension in this specific condition.

The Bayesian correlation analysis between SB-SS Δ_T_ and SB-SS *d*′ (see Supplementary Fig. 4B) confirmed a positive relation between prehension variation and prediction accuracy (*r* = 0.52, *p* = 0.02, *BF*_10_ = 3.52). The BF robustness check indicated moderate evidence in support of the hypothesis about the contribution of similar hand orientation behavior in facilitating a social intention decoding.

## Discussion

Recent literature demonstrated that the kinematics of reach-to-grasp actions performed with different intents is modulated by the underlying motor intention. Interestingly, these modulations seem to be used as a cue to decode other’s intention during action observation^22,33,35^. However, whether the similarity between the observed kinematics and the observer’s own kinematics plays a role in intention recognition is still a matter of debate. To address this issue, we first administered an execution task to identify the kinematic parameters mostly affected by the underlying motor intention. Subsequently, we requested participants to recognize the intention underlying the same reach-to-grasp actions performed by an actor, whose kinematics was recorded as well. The similarity between the key kinematic parameters of the actor and those of the observer significantly contributed to the accuracy in motor intention prediction.

In the execution task, the motor intention underlying the requested actions was varied according to a 2 × 2 factorial design, in which the size of the target (small or big) and the context (social or non-social) where the action occurred were considered as factors. Different kinematic parameters were significantly affected by the two factors, suggesting that different aspects of a motor intention influence different kinematic parameters.

Kinematic components of both reach and grasp were affected by target size, evidencing a wider wrist trajectory and faster grasping movement (wider grip aperture and speeded fingers movement) in the case of a big target. These results are in line with previous studies showing that hand shaping and the transport component are strongly modulated by target size properties^8,54,55^. Overall, we showed that small targets induce more precise and controlled movements, compared to the more ballistic trajectories employed to reach a big target^11^.

The presence of a social target led to a significant decrease in reach velocity and reach deceleration compared to actions targeting a container. In line with the previous results^17,19,32^, arm movements were slower when the action intent was socially oriented, evidencing a more careful and accurate approach when the aim was to pass the object to another person in comparison to place it in an inanimate target container. The result concerning the arm acceleration can be interpreted according to the same principle; indeed, arm acceleration was lower in the social conditions compared to the non-social ones, but only when the target had a big size.

Moreover, to exclude the presence of peculiar hand configuration during the object prehension, we investigated whether differences in hand orientation at the end of the grasping phase occurred among conditions. Participants showed a more lateral prehension when the action required to put the object in the small box relative to the small social condition. Considering that the position of the individual was lateral with respect to the reaching trajectory, we supposed that a more vertical grasping approach could favor a more controlled transport and placing into the hands of another individual, as well as guarantee a higher visibility of the object to the other person.

Results concerning arm acceleration and hand orientation were partially incongruent between the two social tasks. This could be ascribed to the precision required by the different target sizes among conditions. Indeed, actions performed toward a small target require a higher precision to carefully control the placing movement. In this case, modulations of the acceleration could be less evident since the movement is constrained to be slower and more controlled per se. On the contrary, in the case of big targets, the object prehension might result less stable within condition and/or less distinguishable between conditions, as an overall lower precision is required.

Summing up, we confirmed that when we execute an action, different aspects of the underlying motor intention modulate selectively different movement features, giving rise to distinct kinematic patterns. In turn, this indicates that movement planning and motor control processes are attuned to several concurrent factors, including properties of the stimulus (i.e., target size), and the presence/absence of a social interaction.

In the observation task, we requested participants to observe the initial part of the same actions previously executed, i.e. reach-to-grasp, performed by an actor and to predict the motor intention. A recognition rate above chance would imply that different kinematic patterns are somewhat used as cues to predict the unviewed part of the action. More importantly, we aimed to investigate the relation between the kinematic similarity of observed/executed actions and intention recognition accuracy.

Accuracy results showed that observers are able to decode the intention of the observed actions above chance level. However, some differences concerning the accuracy values emerged in the comparison between conditions. Participants performed best when they had to discriminate the target size (85%), while lower performance was found when discriminating between social and non-social targets (73% for small, 58% for big conditions). Overall, these findings confirm how the kinematic modulations intrinsic to an observed reach-to-grasp movement are already informative about the motor intention of others. The highest performance achieved in target size discrimination is in line with the largest set of kinematic parameters found as selective to this factor. The different accuracy in social/non-social recognition tasks, both overcoming the chance rate, might be explained by the fact that actions directed at small targets evidenced the presence of additional spatial cues (i.e., hand orientation), thus possibly facilitating the intention discrimination.

The main novel result of our study concerns the finding of a significant correlation between the accuracy in intention prediction and the similarity of the kinematic profile of the observed action with the observer’s own motor repertoire. The similarity in grasp aperture correlated with the accuracy in target size prediction, while the similarity in reaching acceleration was associated to the accuracy in detecting a social intent.

The pattern of correlations for the social factor was weaker compared to the size factor, since we found a relationship with RMSE, but not with R^2^. This was in line with the lower performance achieved in recognizing a social intention. This could be ascribed to the high variability, proper of temporal parameters such as velocity and acceleration, characterizing the reach-to-grasp kinematics oriented to a social intent. However, it is worth noting that RMSE had a negative relationship with subjects’ accuracy, suggesting how the participants with less variable motor pattern in comparison with the actor kinematics (i.e., lower RMSE) had in general better performances in intention discrimination.

Last, even if we cannot perform a similarity analysis on the hand orientation parameter, the significant positive correlation with accuracy in SmallBox vs. SmallSocial contrast indicated that participants who mostly showed a prehension behavior coherent with the actor better predicted the final intention of the action.

A limitation of our study concerns the absence of a systematic eye tracking during the observation task, and thus of a control of the visual attention level (see^56^). However, excluding trials with too early or too late RTs (see Methods) and inserting catch trials in the experimental design likely has tempered the impact of this lack onto our results.

In summary, the kinematic similarity exerts a facilitative role on the performance in intention recognition, but at the same time we can speculate that this happens only when the relative kinematic patterns across the to-be-discriminated actions are preserved. In presence of opposite patterns, instead, any attempt to match the observed actions with internal templates would contribute confounding information, leading to overall lower accuracy rates.

To our knowledge, our study represents the first behavioral evidence that during action observation, low-level motor features provide cues for decoding the intentions of others, and more importantly, that their effect depends upon how similar they are to the observer’s own kinematics. This result adds new information about the key elements coming into play in the process of action recognition.

Previous research showed that actions belonging to the motor repertoire of the observer are directly mapped on the observer’s motor system, while actions that do not belong to this repertoire appear to be recognized using different circuits, and essentially coded in visual areas^57^. Even within actions present in the observer’s motor repertoire, however, the motor resonance induced by action observation is modulated according to the observer’s motor expertise, being higher for actions more experienced^58^. In connection with this, higher motor resonance appears to be directly related to better action recognition performance^37,59^.

Even if these studies focused on the variation of motor activation in relation to the “what” of the action (in the case of our study, a reach-to-grasp movement) instead of the “how” (the kinematic features expressed by the reach-to-grasp movement), recent evidence showed that motor resonance is sensitive even to subtle differences in movement kinematics characterizing similar actions performed with different intentions^60,61^. Taking together previous studies and our results, we thus speculate that kinematic similarity induces a higher motor resonance, in turn favoring a more accurate motor intention recognition. Indeed, since from the initial phase of an action observation, peculiar kinematic features are detected allowing the observer “to attribute immediately an intentional meaning to the movements of others”^4^. This view reinforces the notion of motor intention recognition as a visuo-motor process, where not only the visual discriminability of the stimuli (see^35^), but also the resonance with the low-level features of the observer motor repertoire plays a fundamental role.

One could argue that we are more accurate in recognizing actions performed with our own kinematics, as this is the kinematics we are most visually exposed to. However, previous studies assessed the individual contribution of visual and motor familiarity in instantiating a motor resonance in the observer’s brain, highlighting that it is the motor experience of a given action which sustains the motor activation during action observation^36^.

Recent neurophysiological studies demonstrated how action perception is coded by the fronto-parietal network involved in mirror mechanism, which resulted to be attuned also to kinematic properties of the actions^24–27,29,62,63^. Within this network, the inferior parietal lobule (IPL) was demonstrated to be responsive to intention representation based on movement kinematics^28^, confirming previous evidence about the role of parietal areas in motor intention coding^64–66^.

Starting from this premise, future studies can approach the study of intention recognition by combining kinematics with neurophysiological techniques, in order to characterize the brain signature of the kinematics “attuning” process in action prediction. While electrophysiological investigations could localize the network recruited by motor intention recognition and describe its internal temporal dynamics^67^, parallel perturbation studies (e.g. repetitive TMS) on the action observation network could unravel the role of each node within the motor intention recognition process. Such studies could also reveal biomarkers specific for the activation of motor areas versus higher order areas involved in inferential processes of mental states of others (e.g., the mentalizing system, see^17^).

## Supplementary information

Supplementary material S1-S4.

## Data Availability

The data that support the findings of this study are available from the corresponding author upon reasonable request.
