# Supplementary material for: Observer-Agent Kinematic Similarity Facilitates Action Intention Decoding
Source: Sci Rep. 2020 Feb 13;10:2605. doi: 10.1038/s41598-020-59176-z (PMC7018748; doi:10.1038/s41598-020-59176-z)
Supplement: Supplementary file 1 — Supplementary material S1-S4. [file 41598_2020_59176_MOESM1_ESM.pdf]

OBSERVER-AGENT KINEMATIC SIMILARITY FACILITATES ACTION INTENTION DECODING

Doriana De Marco, Emilia Scalona, Maria Chiara Bazzini, Pietro Avanzini, Maddalena Fabbri-Destro

SUPPLEMENTARY MATERIAL S1-S2-S3-S4

	NON-SOCIAL (BOX)				SOCIAL (HANDS)			
	**Big Box**		**Small Box**		**Big Social**		**Small Social**	
**REACH**	MEAN	SE	MEAN	SE	MEAN	SE	MEAN	SE
**Amplitude (mm)**	471	5	460	3	464	4	451	4
**Elevation Peak (mm)**	200	3	191	2	192	2	186	3
**Elevation ROM (mm)**	162	3	153	2	153	2	148	3
**Curvature ROM (mm)**	93	2	88	2	89	3	80	2
**Velocity Mean (mm/s)**	624	8	506	14	591	6	540	5
**Velocity Peak (mm/s)**	1029	12	852	23	1011	8	947	11
**Velocity ROM (mm/s)**	977	11	804	22	964	8	900	11
**Acceleration Mean (mm/s^2)**	2464	45	1745	98	2357	73	2200	59
**Acceleration Peak (mm/s^2)**	4861	117	3338	217	4294	151	3884	135
**Acceleration ROM (mm/s^2)**	4838	118	3347	190	4237	141	3855	138
**Deceleration Mean (mm/s^2)**	2685	99	1767	89	2527	65	2066	47
**Deceleration Peak (mm/s^2)**	4263	163	2857	144	4098	143	3495	125
**Deceleration ROM (mm/s^2)**	4285	176	2893	165	4156	161	3525	128
**GRASP**	MEAN	SE	MEAN	SE	MEAN	SE	MEAN	SE
**Finger Aperture Peak (mm)**	75	1	67	1	76	2	70	1
**Finger Aperture ROM (mm)**	48	1	39	1	49	2	43	1
**Finger Velocity Mean (mm/s)**	101	3	60	3	92	5	73	3
**Finger Velocity Peak (mm/s)**	287	13	170	9	281	17	186	10
**Finger Velocity ROM (mm/s)**	284	13	169	9	279	17	184	10
**Acceleration Mean (mm/s^2)**	1717	55	927	49	1479	69	1098	41
**Acceleration Peak (mm/s^2)**	7051	456	4244	315	7132	413	4657	403
**Acceleration ROM (mm/s^2)**	7016	453	4226	315	7121	411	4638	403

Table S1. Mean values and SE of the Reach and Grasp parameters calculated from the reference subject movement. Only the parameters which were significantly affected from CONTEX and SIZE factors are reported.

	NON-SOCIAL (BOX)				SOCIAL (HANDS)			
	**Big Box**		**Small Box**		**Big Social**		**Small Social**	
**REACH**	MEAN	SE	MEAN	SE	MEAN	SE	MEAN	SE
**Amplitude (mm)**	436	10	435	10	432	9	428	9
**Velocity Peak (mm/s)**	1077	40	1069	40	1047	37	1049	38
**Velocity ROM (mm/s)**	1029	40	1021	40	998	37	999	37
**Acceleration Mean (mm/s^2)**	3030	180	2969	185	2903	188	2959	193
**Deceleration Peak (mm/s^2)**	4627	306	4625	297	4405	294	4468	302
**Deceleration ROM (mm/s^2)**	4814	348	4729	308	4504	304	4564	317
**GRASP**	MEAN	SE	MEAN	SE	MEAN	SE	MEAN	SE
**Finger Aperture Peak (mm)**	75	2	75	2	75	2	74	2
**Finger Aperture ROM (mm)**	45	2	44	2	45	2	44	2
**Finger Velocity Mean (mm/s)**	102	6	98	5	101	5	99	5

Table S2. Mean values and SE of the Reach and Grasp parameters calculated from all the 21 participants movements. Only the parameters which were significantly affected from CONTEX and SIZE factors are reported.

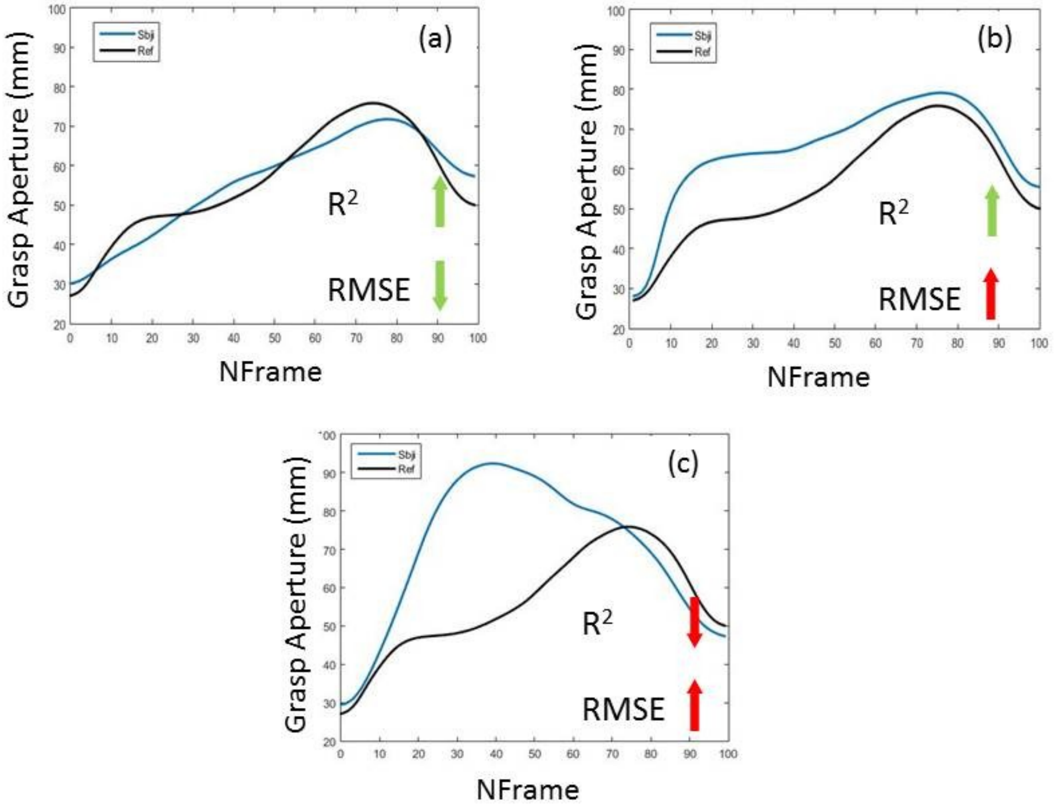

Figure S3. Exemplar combinations of R^2 and RMSE distribution. Panel A reports two highly similar profile, returning high R^2 and low RMSE values. Panel B reports two profiles following a similar pattern, but detaching one from the other, thus maintaining a high R^2 values, but a higher RMSE value. Finally, Panel C reports two profiles highly different both in amplitude and in temporal pattern. This is the worst case, in which R^2 is low and RMSE is high.

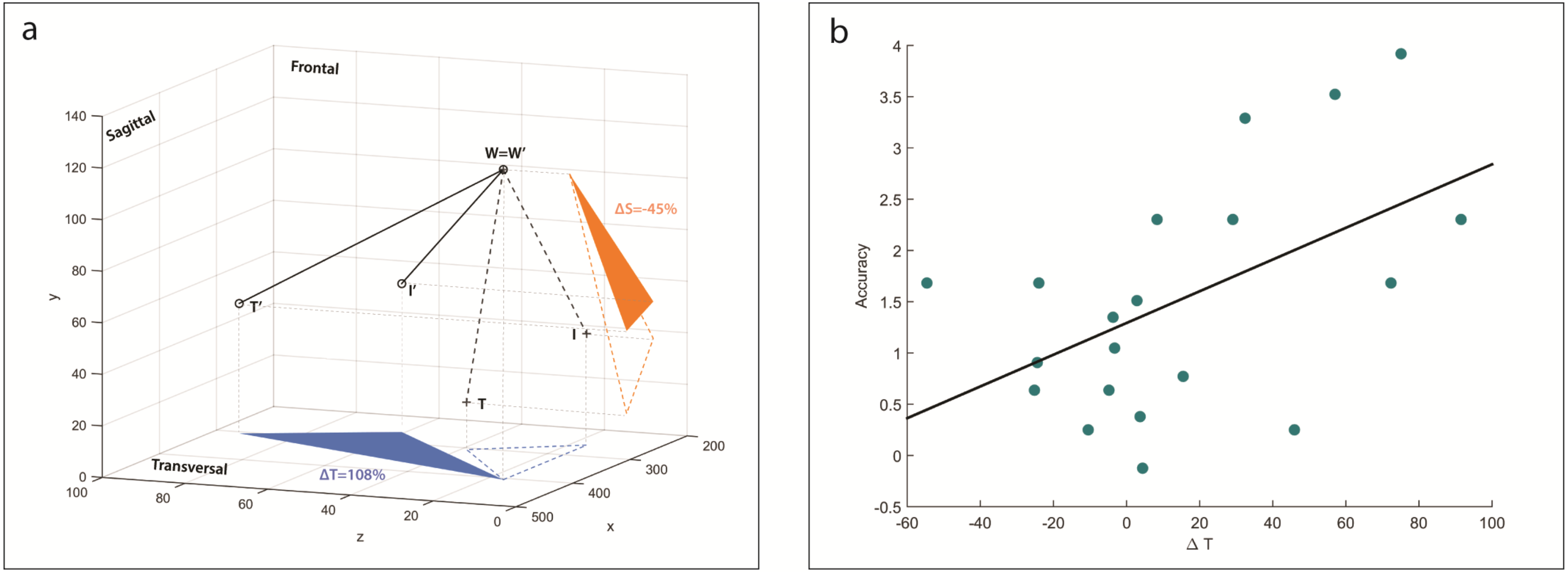

Figure S4: **(a)** example of two different prehensions, one longitudinal and one more lateral, estimated by the wrist, thumb and index markers (W, I and T) and seen from an egocentric perspective. Dashed lines represent the longitudinal prehension; the solid lines represent the more lateral position. The orange triangles represent the projection on the sagittal plane (xy) of the hand, while the blue triangles are the projection on the transversal plane (xz). ΔS and ΔT are the percentage difference between the area evaluated in the two position for the sagittal and trasversal plane, respectively. When moving from a longitudinal to a more lateral position, ΔS is reduced, while ΔT increased. **(b)** Correlation between ΔT relative to the SB-SS condition and recognition accuracy ($r=0.52$; $p=0.02$).
